# Network-Guided Discovery of Influenza Virus Replication Host Factors

**DOI:** 10.1128/mBio.02002-18

**Published:** 2018-12-18

**Authors:** Emily E. Ackerman, Eiryo Kawakami, Manami Katoh, Tokiko Watanabe, Shinji Watanabe, Yuriko Tomita, Tiago J. Lopes, Yukiko Matsuoka, Hiroaki Kitano, Jason E. Shoemaker, Yoshihiro Kawaoka

**Affiliations:** a Department of Chemical & Petroleum Engineering, Swanson School of Engineering, University of Pittsburgh, Pittsburgh, Pennsylvania, USA; b Division of Virology, Department of Microbiology and Immunology, Institute of Medical Science, University of Tokyo, Tokyo, Japan; c ERATO Infection-Induced Host Responses Project, Japan Science and Technology Agency, Saitama, Japan; d The Systems Biology Institute, Tokyo, Japan; e Department of Pathobiological Sciences, School of Veterinary Medicine, University of Wisconsin—Madison, Madison, Wisconsin, USA; f Laboratory for Disease Systems Modeling, RIKEN Center for Integrative Medical Sciences, Yokohama, Kanagawa, Japan; g Okinawa Institute of Science and Technology, Okinawa, Japan; h Department of Computational and Systems Biology, School of Medicine, University of Pittsburgh, Pittsburgh, Pennsylvania, USA; i Department of Special Pathogens, International Research Center for Infectious Diseases, Institute of Medical Science, University of Tokyo, Tokyo, Japan; Fred Hutchinson Cancer Research Center; University of Washington

**Keywords:** drug targets, influenza, protein-protein interactions, virus-host interactions

## Abstract

The positions of host factors required for viral replication within a human protein-protein interaction (PPI) network can be exploited to identify drug targets that are robust to drug-mediated selective pressure. Host factors can physically interact with viral proteins, be a component of virus-regulated pathways (where proteins do not interact with viral proteins), or be required for viral replication but unregulated by viruses. Here, we demonstrate a method of combining human PPI networks with virus-host PPI data to improve antiviral drug discovery for influenza viruses by identifying target host proteins. Analysis shows that influenza virus proteins physically interact with host proteins in network positions significant for information flow, even after the removal of known abundance-degree bias within PPI data. We have isolated a subnetwork of the human PPI network that connects virus-interacting host proteins to host factors that are important for influenza virus replication without physically interacting with viral proteins. The subnetwork is enriched for signaling and immune processes distinct from those associated with virus-interacting proteins. Selecting proteins based on subnetwork topology, we performed an siRNA screen to determine whether the subnetwork was enriched for virus replication host factors and whether network position within the subnetwork offers an advantage in prioritization of drug targets to control influenza virus replication. We found that the subnetwork is highly enriched for target host proteins—more so than the set of host factors that physically interact with viral proteins. Our findings demonstrate that network positions are a powerful predictor to guide antiviral drug candidate prioritization.

## Abstract (precis)

Integrating virus-host interactions with host protein-protein interactions, we have created a method using these established network practices to identify host factors (i.e., proteins) that are likely candidates for antiviral drug targeting. We demonstrate that interaction cascades between host proteins that directly interact with viral proteins and host factors that are important to influenza virus replication are enriched for signaling and immune processes. Additionally, we show that host proteins that interact with viral proteins are in network locations of power. Finally, we demonstrate a new network methodology to predict novel host factors and validate predictions with an siRNA screen. Our results show that integrating virus-host proteins interactions is useful in the identification of antiviral drug target candidates.

## INTRODUCTION

Viruses such as influenza virus hijack and reprogram host cellular machinery to perform virus replication tasks. Influenza outbreaks have a major impact on public health and the global economy each year (1, 2). While annual vaccinations provide some protection, vaccination effectiveness is impaired by antigenic drift and availability issues (3, 4). Recent sporadic human infections with avian viruses of H5N1 and H7N9 subtypes have raised concerns about the pandemic potential of these viruses (5 – 8). Antiviral drugs that target influenza viral proteins are available (9, 10), but drug-resistant strains have emerged (11, 12). Therefore, there is an urgent need to identify drug targets that are robust to virus mutation and drug-mediated selective pressure.

**FIG 1:**
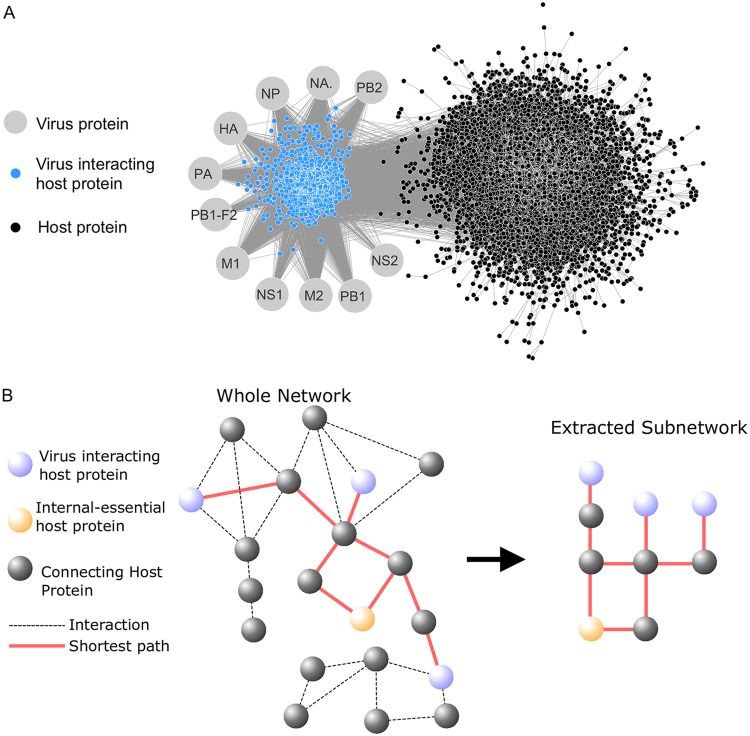
The virus-interacting network and the virus subnetwork. (A) The virus-interacting network is created from human host-PPI data combined with virus-host protein interaction data. (B) The virus subnetwork was isolated from the complete human PPI network by collecting all interactions involved in the shortest paths (red) that connect influenza virus-interacting proteins (blue) to human proteins essential to virus replication (e.g., the internal-essential proteins) (orange). The connecting proteins (black) are candidates to be evaluated for their antiviral properties.

Understanding virus-host interactions in the context of the human protein-protein interaction (PPI) network will provide a global perspective into how influenza virus manipulates host proteins and aid in identifying host factors involved in influenza virus replication for drug targeting (13 – 15). The virus-host interactome is visualized in Fig. 1A. Within a PPI network, a protein’s global significance can be assessed by the protein’s network centrality, the identification of important components based on information flow across the network. Common measures include a protein degree (number of binding partners) and betweenness (the degree to which the protein is a bottleneck in the network) though several others exist (16, 17). Network centrality has emerged as a valuable tool for studying proteins associated with cancer (18, 19) and drug targeting (19 – 22). PPI network-based approaches have recently been utilized in influenza virus studies to identify and study potential factors involved in virus replication (23 – 27). Network studies have demonstrated that virus-interacting host proteins tend to have a high network significance based on a variety of network metrics (including betweenness and degree) for several viruses, including influenza viruses (28) and hepatitis C virus (29). A comparative analysis of influenza virus protein and host protein interactomes has identified novel host factors that are common across the protein interactomes (30). Furthermore, meta-analysis studies have developed statistical methods to better identify host factors by leveraging data from several virus replication screens (31). However, how effectively can virus-host protein interaction data and network topology be leveraged to improve host factor identification (i.e., antiviral drug target identification) remains a question.

Here, we demonstrate a method of integrating virus-host protein interaction data into a human PPI network to prioritize host proteins as antiviral drug target candidates. First, we analyzed a set of 1,292 human proteins identified previously as having interactions with influenza virus proteins (32), 299 of which were found to significantly inhibit influenza virus replication during an siRNA virus replication screen (Fig. 1A). Consistent with previous studies, we show that virus-interacting human proteins tend to be in positions essential to PPI network information flow and are closely clustered within the PPI network. We then isolated the subnetwork of the human PPI network that connects virus-interacting host proteins to noninteracting host factors (referred to as “internal”) that were identified to be important for influenza virus replication in a study and reevaluated in this work (33) (Fig. 1B). Candidate proteins connecting virus-interacting host proteins to internal host factors were selected based on their betweenness within this subnetwork and evaluated by viral replication screen. Betweenness was selected under the hypothesis that selecting network bottlenecks (i.e., high-betweenness proteins) would limit the opportunity for the virus to engage host machinery through alternative pathways. The fraction of proteins tested that significantly reduced virus replication (i.e., the hit rate) was compared to the hit rate observed in a genome-wide screen, the hit rate when screening virus-interacting proteins (the virus’ nearest neighbors in the network) and the hit rate observed when screening host factors identified in a previous study (33).

## RESULTS

### Host proteins that interact with influenza virus proteins are central to the PPI network.

Studies have shown that proteins in network positions that are essential for information flow within a PPI network (e.g., high degree or high betweenness) are more likely to be associated with diseases (34, 35) or drugs with known, dangerous side effects (19, 36). Using a human PPI network, we analyzed the network topology characteristics of virus-interacting and non-virus-interacting host proteins. In a previous study, we identified 1,292 host proteins that coprecipitated with at least one of 11 influenza virus proteins (viral PB2, PB1, PA, HA, NP, NA, M1, M2, NS1, NS2, and PB1-F2 proteins) (32). These proteins are referred to as “virus-interacting proteins.” We mapped the interaction data onto a human PPI network developed from the Human Integrated Protein-Protein Interaction rEference (HIPPIE) database (37). After constraining the network to highly confident interactions (see Materials and Methods), the PPI consisted of one large network (9,969 proteins and 57,615 interactions) which contained 1,213 influenza virus-interacting host proteins and 86 smaller networks that contained 7 or fewer proteins (the majority containing only 2 proteins) and no influenza virus-interacting proteins. The smaller networks were removed from further consideration.

**FIG 2:**
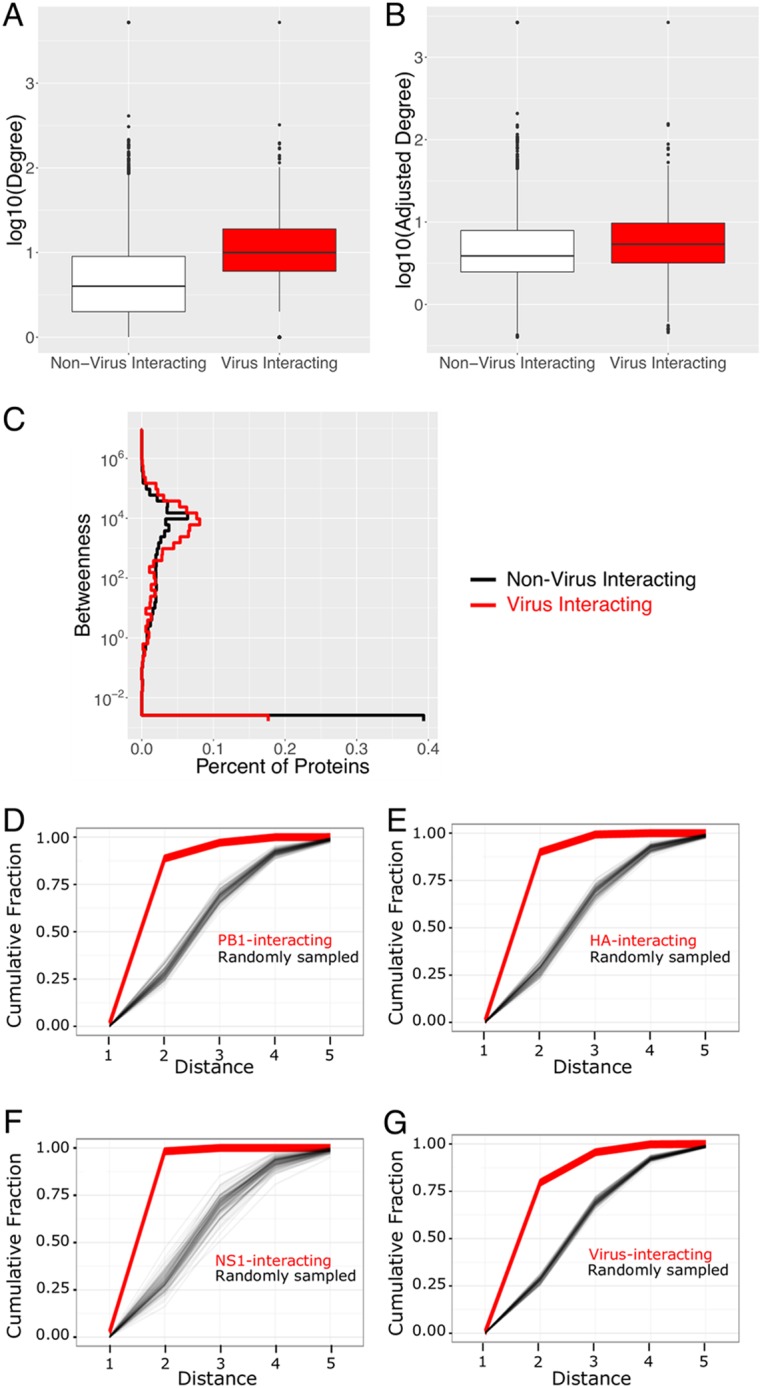
The network topological characteristics of virus-interacting host proteins. (A to C) Distributions of the degree (A), adjusted degree (B), and betweenness (C) of virus-interacting proteins and all proteins in the human PPI network. An ε of 0.01 was added to the betweenness to facilitate log scaling. (D to G) The cumulative distributions (thick red lines) of the shortest distances connecting host proteins in the PPI network that interact with viral PB1 (D), HA (E), or NS1 (F) protein or the set of all viral proteins (G). For a control, the cumulative distribution of distances was iteratively determined (*N* = 100) by randomly sampled host proteins in the PPI network (thin black lines). The number of proteins sampled on each iteration was equal to the number of interacting host proteins of each virus protein (or set of viral proteins).

Virus proteins were significantly more likely to interact with host proteins that were in positions of high regulatory importance in the human PPI network. For every protein, the degree (number of neighbor proteins) and betweenness (38) (measure of the shortest paths between all other proteins in the network that include the protein in question) were calculated. On average, the degrees of virus-interacting host proteins were twice the degrees of all proteins and significantly higher than the degrees of the non-virus-interacting proteins of the network (Fig. 2A; median degrees of virus-interacting, non-virus-interacting, and all proteins = 10, 4, and 5, respectively; Student *t* test *P* value for comparing log-scaled non-virus-interacting and virus-interacting data < 10^−16^).

Knowing documented bias between protein abundance and protein degree (39), an analysis of the correlation between the two variables was performed to ensure the high degree of virus-interacting proteins was not biased by protein abundance. Significant correlation exists between abundance and degree in the virus-interacting proteins (Pearson correlation coefficient, 0.23; *P* value, 1.2 × 10^−13^), subnetwork (Pearson correlation coefficient, 0.10; *P* value, 6.9 × 10^−7^), and the total network (Pearson correlation coefficient, 0.25; *P* value, 2.2 × 10^−16^). After fitting degree and abundance to a linear model, the bias due to abundance was removed (see Materials and Methods; Pearson correlation coefficient after adjustment, −0.07; adjusted degree values can be found in Table S1 in the supplemental material; Fig. S1 demonstrates the adjusted degree versus abundance). A comparison of the adjusted degrees of the non-virus-interacting and virus-interacting proteins reveals that the previous conclusions remain: the degree of virus-interacting proteins is significantly higher than the degree of non-virus-interacting proteins in the network (Fig. 2B; median degree of virus-interacting, non-virus-interacting, and all proteins = 5.4, 3.9, and 4.2, respectively; Student *t* test *P* value for comparing log-scaled non-virus-interacting and virus-interacting data < 10^−16^). Therefore, despite known biases ingrained in PPI data, virus-interacting proteins bind more proteins than non-virus-interacting proteins.

10.1128/mBio.02002-18.1 FIG S1 Protein abundance versus protein degree for the total network’s original degree (A) and adjusted degree (B). Each plot is fit with a linear model in red, demonstrating that the correlation found in the original degree is not present after value adjustment. Download FIG S1, TIF file, 1.2 MB. Copyright © 2018 Ackerman et al. 2018 Ackerman et al. This content is distributed under the terms of the Creative Commons Attribution 4.0 International license.

10.1128/mBio.02002-18.5 TABLE S1 Degree, betweenness, and abundance data for all proteins in the PPI network. Each protein (listed by Gene ID, symbol, and description) is listed with degree, betweenness, and virus protein binding partner information (1 for binds virus protein, 0 for does not bind virus protein). Sheet A shows the total network and also contains abundance data from Geiger et al. (64), and adjusted degree. Sheet B shows the subnetwork and also contains degree and betweenness in the subnetwork and identifies which proteins were selected for further testing. Download Table S1, XLSX file, 0.8 MB. Copyright © 2018 Ackerman et al. 2018 Ackerman et al. This content is distributed under the terms of the Creative Commons Attribution 4.0 International license.

Virus-interacting proteins also had a significantly higher betweenness (Fig. 2C; median betweenness of virus-interacting and all proteins = 1,625.1 and 32.8, respectively; Mann-Whitney U test *P* value for log-scaled data < 10^−16^). Comparing median betweenness after the removal of proteins with a betweenness of zero, virus-interacting proteins still had a significantly higher betweenness though the population medians were closer in value (median betweenness of virus-interacting and all proteins = 3,981.1 and 1,584.8, respectively; Mann-Whitney U test *P* value for the log-scaled data = 8.2 × 10^−16^). The tendency for virus proteins to bind host proteins that had a higher degree and betweenness was consistent when analyzing the interaction partners of each virus protein separately (Fig. S1; pairwise *t* test of the log-scaled data. All *P* values were <0.01 except for betweenness of NS2-interacting proteins, which was not significantly distinct from the betweenness of the full PPI). This indicates that influenza virus proteins selectively interact with human proteins that can strongly regulate cellular behavior. These results are consistent with published findings for HCV and dengue virus (40, 41) and with a previous study which used a yeast two-hybrid approach to identify influenza virus-interacting host proteins for 10 of the 11 virus proteins (28). Further, these are characteristics that generalize to each virus protein’s interacting partner; suggesting that all 11 virus proteins have a role in manipulating cellular machinery.

### Influenza virus-interacting host proteins are closely connected in the human PPI network.

Next, we evaluated whether virus-interacting proteins tend to cluster closely to one another in the PPI network. A previous study suggested that host factors of viral replication are closely clustered within the network but did not assess the topological characteristics of virus-interacting host proteins (42). Functionally related proteins are often observed to be closely clustered in PPI networks (43, 44). Knowing that influenza virus proteins manipulate multiple host cell functions to promote replication, these previous studies suggest that the interaction partners of viral proteins should be closely clustered by host function. If this is true, neighboring cluster proteins could serve as possible alternatives for influenza virus to manipulate each host function.

We quantified how close each virus protein’s interacting host proteins are within the network by calculating the shortest distances required to connect all of the host proteins that interact with a viral protein, creating a distribution of distances. The cumulative distribution details the fraction of host proteins that could be connected to other host proteins that bind the same viral protein in *n* or fewer steps. For a control, we determined the cumulative distribution of distances that result from randomly sampled proteins in the network. For a single iteration, we created a set of random proteins. The size of the set was determined by the number of proteins which interact with the virus protein of interest (e.g., PB1 has 322 interacting host proteins; therefore, 322 proteins were randomly selected from the network; Fig. 2D to G). The distributions of distances connecting all of the randomly sampled proteins was calculated. This process was repeated 100 times.

We found that virus-interacting host proteins are very significantly clustered within the PPI network. The set of proteins that interact with a viral protein are significantly more closely clustered in the network than expected by chance (Fig. 2D to G, *P* < 0.01 comparing the median distance of the virus-interacting proteins to the median distance of randomly sampled proteins). Generally, ∼25% of the randomly sampled proteins are connected by two or fewer interactions, while 88.7% of PB1-interacting proteins, 90.0% of HA-interacting proteins, 98.2% of NS1-interacting proteins, and 79.6% of all host proteins that interact with any influenza virus protein are connected by two or fewer interactions. Collectively, these results support that viral proteins are selectively targeting closely clustered host proteins.

We next evaluated whether influenza virus-interacting proteins are often components of a common protein complex. To do so, we determined the fraction of all influenza virus-interacting protein pairs (735,078 pairs in total) that appear within a protein complex and compared that fraction to the fraction of all protein pairs (49,685,496 total pairs) in the PPI that appear in a protein complex. Mammalian protein complex information was downloaded from CORUM (a comprehensive resource of mammalian protein complex data) (45). We found that 1.5% of all virus-interacting protein pairs are involved in a complex, whereas only 0.066% of all protein pairs in the PPI are involved in a complex. In sum, influenza virus proteins are closely clustered and 22.4 times more likely to be involved in a protein complex than randomly selected proteins.

### Constructing the influenza virus-host subnetwork.

Network analysis of virus-interacting host proteins demonstrates that viral proteins preferentially interact with closely connected host proteins that are in positions central to information flow across the human PPI network, suggesting that it may be possible to exploit network positions to prioritize potential antiviral drug targets. We hypothesized that there exists a subnetwork consisting of pathways that connect virus-interacting proteins to key cellular machinery that is likely to be significantly enriched for host factors. We further hypothesized that the topology of host factors within this subnetwork may provide an additional advantage in identifying host factors.

To evaluate these hypotheses, we first performed an siRNA screen of host factors identified in a previous genome-wide screen for influenza virus host factors to identify key host factors that do not interact directly with the virus (33). Poor repeatability due to differences in the experimental conditions and possibly high false-negative rates (42) often characterizes siRNA screens of influenza virus replication host factors. Here, HEK293 cells were transfected with siRNAs targeting 264 non-virus-interacting host factors identified by Karlas et al. (33) (two siRNAs per gene were used, as shown in Table S2; AllStars Negative Control siRNA [Qiagen] was used as a negative control) and then infected with influenza virus at 24 h posttransfection. The culture supernatants were harvested for virus titration at 48 h postinfection. Virus titers were determined by plaque assay. A protein was defined as a hit if the virus titers decreased by at least two log units upon transfection with an adjusted *P* value of <0.01. The viability of siRNA-transfected cells was assessed using Cell-Titer Glo assay, and downregulation of mRNA levels for the hit proteins was confirmed by quantitative reverse transcription-PCR (qRT-PCR). Of the 264 previously identified host factors tested, 71 significantly downregulated virus replication. Of the 71, 21 were identified to directly interact with influenza virus proteins. In all, 50 of the host factors downregulated virus growth and do not directly interact with the virus. We labeled these proteins as “internal-essential” host factors.

10.1128/mBio.02002-18.6 TABLE S2 Effects of siRNAs targeting host factors identified to be important for influenza virus replication by Karlas et al. (Nature, 2010) on virus production. Note that two siRNAs were used per Entrez Gene ID. Sheet 2, labeled “Untested host factors,” lists host factors that were identified in the Karlas screen but were not evaluated in this study. Sheet 3, labeled “Effect on virus replication,” includes virus titers observed in HEK293 cells. Download Table S2, XLSX file, 0.1 MB. Copyright © 2018 Ackerman et al. 2018 Ackerman et al. This content is distributed under the terms of the Creative Commons Attribution 4.0 International license.

**FIG 3:**
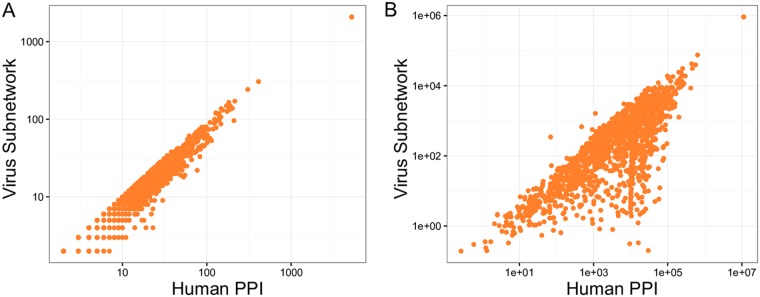
Network characteristics of the virus subnetwork. Panels A and B compare the degree and betweenness, respectively, of the connecting proteins in the whole PPI network and the virus subnetwork.

Next, we constructed an influenza virus-specific subnetwork (process illustrated in Fig. 1B) of the shortest paths connecting virus-interacting host proteins to “internal-essential” host factors (i.e., the host factors reverified in the siRNA screen of host factors identified in the screen of Karlas et al. [33]). The proteins linking internal-essential proteins to virus-interacting proteins are “connecting” candidate proteins for evaluation as host factors of virus replication. The resulting subnetwork contained 1,213 virus-interacting proteins, 38 internal-essential proteins (12 proteins were not in the PPI network), and 1,643 connecting candidate proteins (Table S1 contains the identities and centrality values for all proteins in the subnetwork). As a result of how the subnetwork is constructed, the mean degrees of the virus-interacting proteins and the internal-essential proteins were lower than the mean degree of the connecting proteins (Fig. S2A; ANOVA followed by Tukey *post hoc* analysis *P* < 0.01). While the degree of connecting proteins does not shift significantly between the total PPI network and the virus subnetwork (Fig. 3A), some proteins with low betweenness have much lower betweenness in the virus subnetwork compared to the total PPI network (Fig. 3B). Higher betweenness nodes in the total PPI network do not demonstrate dramatic shifts in the virus subnetwork upon comparison. This shift between the total network and virus subnetwork may reveal proteins that are more or less critical to virus replication which cannot be identified in a standard PPI network analysis.

10.1128/mBio.02002-18.2 FIG S2 The distributions of the degree (a) and betweenness (b) of the interaction partners of each of the 11 virus proteins. The *y* axis lists the particular virus protein, and the *x* axis demonstrates distributions of the centrality measures of the virus protein’s interaction partners within the human PPI network. The distributions for all proteins in the human PPI network (labeled “All”) and the set of proteins that interacted with any of the virus proteins (“VB”) are included for comparison. Download FIG S2, TIF file, 0.3 MB. Copyright © 2018 Ackerman et al. 2018 Ackerman et al. This content is distributed under the terms of the Creative Commons Attribution 4.0 International license.

### Functional enrichment analysis of the influenza virus-host subnetwork.

A functional enrichment analysis was performed using DAVID 6.8’s Functional Annotation tool (46). Previous work (32) included a per protein analysis of virus-interacting proteins that identified involvement in several stages of the life cycle of influenza virus, particularly in viral replication tasks and export of influenza vRNPs from the nucleus. Here, analysis found that virus-interacting host proteins and connecting (non-internal-essential) proteins within the virus subnetwork are functionally distinct (see Tables 1 and 2 for abbreviated results; see Table S3 for full results). Analysis of virus-interacting host proteins replicated the previous finding that virus-interacting host proteins are primarily associated with housekeeping and viral replication processes (32), whereas analysis of connecting proteins shows association with protein phosphorylation, histone reconfiguration, and immune responses. Specifically, the immune response pathways identified are the stimulatory C-type lectin receptor signaling, T-cell receptor signaling, and Fc epsilon receptor signaling, all of which regulate NF-κB activity. These results suggest that the virus subnetwork contains functional information generally unobserved when considering virus-interacting host proteins or internal-essential proteins in isolation.

**TABLE 1:** Functional enrichment analysis of connecting proteins within the virus subnetwork^*a*^

Cluster	No. of GO terms	Enrichment score
Transcription	4	55.4
DNA damage/repair	3	19.2
Protein phosphorylation	19	18.7
Mitosis	5	18.7
Histone reconfiguration	42	14.4
Immune response	3	14.0
C-type lectin receptor signaling pathway		
T-cell receptor signaling pathway		
Zinc ion binding	4	11.5

a Proteins were analyzed using DAVID.

**TABLE 2:** Functional enrichment analysis of virus-interacting proteins within the virus subnetwork^*a*^

Cluster	No. of GO terms	Enrichment score
Ribonucleoprotein/viral transcription	13	67.2
Cell-cell adhesion	3	45.0
mRNA splicing	9	41.8
Nucleotide binding	10	30.3
Chaperone/UPR	3	22.1
Viral nucleocapsid	3	19.0
mRNA nuclear export	4	17.5
Nucleotide binding/ATP binding	5	17.3
Translation initiation factors	11	13.2
Proteasome/NF-κB MAPK signaling	23	12.1

a Proteins were analyzed using DAVID.

10.1128/mBio.02002-18.7 TABLE S3 DAVID functional annotation tool results for virus-interacting proteins and connecting proteins of the influenza virus subnetwork. Full results include the clustering, chart, and table outputs from DAVID 6.8. Download Table S3, XLSX file, 2.3 MB. Copyright © 2018 Ackerman et al. 2018 Ackerman et al. This content is distributed under the terms of the Creative Commons Attribution 4.0 International license.

### Connecting proteins of the influenza virus-host subnetwork are more enriched for host factors than virus-interacting proteins are.

To evaluate the hypothesis that the “connecting” proteins are likely to be host factors and to simultaneously evaluate whether network topology can improve host factor identification, we selected 78 proteins of the subnetwork with the highest (*n* = 39) and lowest betweenness (*n* = 39) and conducted another *in vitro* virus replication assay. HEK293 cells were again transfected with siRNAs targeted to each of the 78 candidate protein’s genes, and the procedure described previously was performed to determine the proportion of the connecting proteins tested that are host factors of influenza virus replication. The hit rate is defined as the proportion of proteins tested that significantly downregulated virus replication.

To evaluate the significance observed in the virus replication screen of the connecting proteins, we compared the observed hit rate to the hit rate observed in a screen of the 1,292 virus-interacting host proteins in HEK293 cells (hit rate = 299/1,292 = 0.23) (32), in the screen of the 264 host factors in the study by Karlas et al. (33) (detailed above), and in a whole-genome screen for influenza virus host factors in A549 cells (287/22,843 = 0.013) (33). The whole-genome screen provides the expected hit rate when randomly sampling the PPI. An alternative approach to network-based discovery is to target the nearest neighbors of the virus, a comparison provided by screening virus-interacting host proteins. An additional metric is the hit rate observed in our siRNA screen of the host factors identified by Karlas et al. (33) (71 out of 264; hit rate = 0.27). Differences between hit rates were compared using Pearson’s chi-squared test when comparing proportions between two binomial groups.

**FIG 4:**
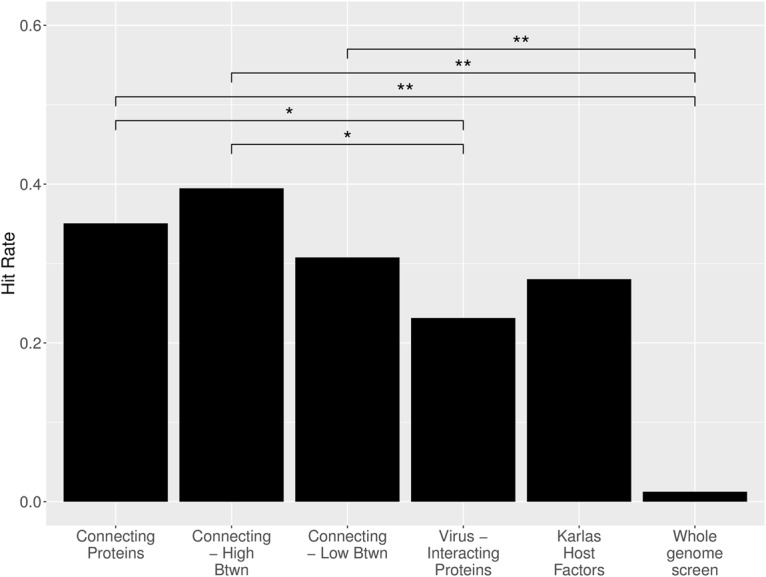
Comparison of hit rates. The hit rates are reported for all tested connecting proteins (proteins linking internal-essential proteins to virus-interacting proteins) and the subset of connecting proteins with the highest and lowest betweenness in the virus subnetwork. These hit rates are compared to hit rates observed from a previous screen of virus-interacting host proteins (labeled “Virus-Interacting Proteins”) (32), from applying our screening methodology to host factors identified in a screen by Karlas et al. (33) (labeled “Karlas Host Factors”) and from a genome-wide screen (33). Prop.test in R was used to determine the significance of the difference in hit rates observed for binomial groups. Values that are significantly different are indicated by bars and asterisks as follows: *, *P* < 0.05; **, *P* < 0.01.

The siRNA screen of the connecting proteins found that connecting proteins were significantly enriched for host factors, but there was no statistically significant advantage in selecting proteins by betweenness (Fig. 4). Of the 78 proteins targeted in the siRNA screen of connecting proteins, a total of 27 significantly reduced virus titers by at least 2 orders of magnitude, corresponding to 15 categorized as connecting high-betweenness proteins and 12 categorized as connecting low-betweenness proteins. Note that one of the 39 connecting high-betweenness proteins (PLK1) was eliminated from the calculation because both respective siRNAs were cytotoxic (Table S4). The hit rate of connecting proteins (27/77 = 0.35) was significantly higher than the hit rate observed in the screen of virus-interacting proteins (*P* = 0.024) and in the whole-genome screen (*P* < 2.2 × 10^−16^) but not significantly distinct from the rate observed in rescreening the Karlas host factors (*P* = 0.21). When considering the connecting proteins based on their betweenness, the high-betweenness connecting proteins had a hit rate of 0.39 (15/38) which was significantly higher than the hit rates observed in the virus-interacting and whole-genome screens (*P* = 0.032 and *P* < 2.2 × 10^−16^, respectively). The high-betweenness protein hit rate was higher than the rate observed in the screen of host factors by Karlas et al. (33), but not significantly (*P* = 0.16). The low-betweenness connecting protein hit rate was lower than that of the high-betweenness connecting proteins (12/39 = 0.31). The difference in hit rates between high- and low-betweenness proteins was not significant (*P* = 0.57). In all, the screening results suggest that proteins connecting virus-interacting proteins to host factors of influenza virus replication are highly enriched for host factors themselves—significantly more so than proteins that directly interact with virus proteins. However, the topological information from betweenness does not significantly improve host factor identification.

10.1128/mBio.02002-18.8 TABLE S4 Effects of siRNAs targeting host factors with high or low betweenness in the virus-host subnetwork on virus production. Download Table S4, XLSX file, 0.02 MB. Copyright © 2018 Ackerman et al. 2018 Ackerman et al. This content is distributed under the terms of the Creative Commons Attribution 4.0 International license.

### The influenza virus subnetwork is enriched for host factors identified in six host factor screens.

To determine whether host factors identified in previous screens are enriched within the virus subnetwork, we compiled a list of host factors of influenza virus replication identified in at least one of six previous screens (33, 47 – 51) (Table S5). A Fisher exact test for enrichment was used to determine whether the connecting proteins or the set of influenza virus-interacting proteins are enriched with host factors identified in these studies relative to the abundance of host factors within the PPI network. Both connecting proteins and the virus-interacting proteins are significantly enriched for host factors (*P* = 7.2 × 10^−05^ and *P* = 1.1 × 10^−05^, respectively; odds ratio = 1.4 and 1.5, respectively). There is no significant difference in the enrichment of host factors between connecting proteins and virus-interacting proteins (*P* = 0.48; odds ratio = 0.92). To ensure the host factors identified in the study of Karlas et al. (33) were not creating bias in the enrichment result, the enrichment analysis was repeated using host factors identified in all studies except the Karlas study. Again, connecting proteins and virus-interacting proteins are significantly enriched for host factors (*P* = 1.8 × 10^−06^ and *P* = 3.2 × 10^−03^, respectively; odds ratio = 1.5 and 1.34, respectively), and no significant difference in the enrichment of host factors between connecting proteins and virus-interacting proteins was found (*P* = 0.49).

10.1128/mBio.02002-18.9 TABLE S5 Hit lists of genes identified in six independent genome-wide screens. Studies include König et al. (2010), Brass et al. (2009), Shapira et al. (2009), Hao et al. (2008), Karlas et al. (2010), and Sui et al. (2009). Download Table S5, XLS file, 0.1 MB. Copyright © 2018 Ackerman et al. 2018 Ackerman et al. This content is distributed under the terms of the Creative Commons Attribution 4.0 International license.

## DISCUSSION

Network approaches have demonstrated their potential impact on health-related research, including gene/protein characterization and drug design and side effects (14, 18, 19, 22, 36, 52), yet demonstrations that network information can inform drug target discovery are still limited. Here, we present the first confirmation that virus and host protein interaction data can be integrated to improve large-scale drug target discovery (specifically antiviral target discovery) and reveal additional insights into virus-host interactions. The positions of virus-interacting proteins suggest that the influenza virus has evolved to interact with proteins that influence network behavior, regardless of known abundance-degree biases in PPI data generation (which has not previously been demonstrated). The virus-specific subnetwork reveals that there is a set of proteins with moderately high betweenness in the total network yet low betweenness in the subnetwork. While these proteins may be of high importance to the total network, this result suggests that they may be less important to the progression of influenza infection than proteins which are of high betweenness to both the total network and virus-specific subnetwork. In this way, the novel subnetwork construction provides further insight when determining important host factors of virus replication.

Virus-interacting proteins are closely clustered in the network, which may be a result of attempts by the virus to manipulate specific biological functions (as proteins with shared biological functions tend to cluster in PPI networks [53]). This may signify that influenza virus has parallel available pathways to engage with host biological functions. Additionally, complex evidence suggests that high degree and high clustering of IAV proteins may be due to their involvement in protein complexes. From a network viewpoint, it is likely that high incidence of clustering within the PPI network is a result of both the high betweenness and degree of the virus-interacting protein group as a whole. Previous studies have found that host factors of virus replication (not necessarily virus-interacting host proteins) have been observed to cluster within the PPI network (42). Further analysis on network clustering host factors of interest is needed to determine whether these two observations are independent of one another.

Functional enrichment analysis of the subnetwork reinforces that while virus-interacting proteins are associated with virus replication processes, proteins within the constructed subnetwork are associated with immune response to viral infection. Results for virus-interacting proteins largely build on the per protein discussion of virus-host interactions found in previous work (32), identifying involvement in several stages of the viral replication cycle. The functional enrichment analysis of connecting proteins reveals high levels of involvement in the immune response to viral infection, specifically in NF-κB regulating pathways such as stimulatory C-type lectin receptor signaling, T-cell receptor signaling, and Fc epsilon receptor signaling. Influenza virus is known to manipulate host immune response pathways (specifically NF-κB regulating pathways) to promote viral replication (54, 55). Because previous virus-host PPI network analyses have not studied these connecting proteins as a separate population, their importance to the biology and regulation of the system has been overlooked. The subnetwork construction approach applied in this work isolates additional host biological processes essential to the regulation of virus replication, further demonstrated by siRNA screening results of the connecting proteins. Together, the results suggest that future work in virus-host protein networks can leverage the technique presented here to identify subnetworks with increased biological relevance to the analyzed phenotypes/conditions and increase predictive power for the purposes of drug discovery.

The conclusion that host-virus interaction data can be leveraged to improve virus replication host factor discovery is unlikely to be affected by off-target concerns associated with siRNA screens. Though off-target concerns often challenge siRNA studies, changes to experimental protocols (such as requiring multiple siRNA hits per targeted gene or changing siRNA concentrations) can only moderately improve false-positive rates (56 – 58). The protocol used in this study was not optimal to ensure the characterization of any one targeted gene. As such, the hit rates of gene groups are compared. Protocols between these experiments and those used for comparisons are either identical (32) or very similar (33), suggesting that off-target rates across the tested groups are unlikely to explain the differences in observed hit rates.

The variability and incompleteness of PPI data as well as the limited agreement between influenza virus replication screens are well-known concerns for network-based drug target discovery. False discovery of virus-host interactions and the possibility that virus-host interaction data are skewed toward well-studied networks could also have an effect on the clustering result in virus-interacting proteins. However, the enrichment of host proteins important for influenza virus replication within the constructed virus subnetwork demonstrates that even with these possible shortcomings, PPI network analyses have the power to identify important host factors for influenza virus replication. The antiviral protein candidate screen performed in this study further supports the use of PPI data during candidate prioritization with significant hit rates against virus-interacting proteins and randomly targeted proteins.

The observation that betweenness does not significantly improve host factor prediction suggests that alternative topology measures should be considered. There were several reasons why betweenness was selected. Biological pathways are known to have several alternative routes to maintaining cellular operations, a key feature of biological robustness (59 – 61). Biological networks are also theorized to have a bow tie-like structure that suggests a natural bottlenecking within the PPI network near critical cellular machinery (62). These concepts together suggest targeting bottlenecks (high-betweenness proteins) as a means of mitigating escape via alternative paths. It is also a concern for alternative pathways as to why the set of virus-interacting proteins was not limited to confirmed host factors of influenza virus replication. In future work, other network topology measures (e.g., degree, Burt’s constraint, or closeness) could be tested in the subnetwork and subnetwork construction and could be varied to consider different subsets of either the virus-interacting proteins or the internal host factors. Even so, the results suggest that construction of the virus-specific subnetwork provides major advantages in host factor discovery and can significantly expand drug candidate repertoires beyond virus-interacting proteins. Furthermore, since the connecting proteins do not directly interact with the virus, they may be more resistant to concerns related to drug-mediated selective pressure.

Another interesting continuation of this study would identify the cause of the effect of connecting proteins on virus replication. The mechanism by which each host factor is regulating virus replication may offer additional clues for drug candidate prioritization efforts. Overall, this PPI-based study provides insight into the network characteristics of virus-host interactions and supports the idea that the influenza virus evolved to interact with host proteins in dominant network positions in order to maximally manipulate host cells.

## MATERIALS AND METHODS

### Protein-protein interaction network construction and analysis.

Protein-protein interaction (PPI) data were downloaded from the Human Integrated Protein-Protein Interaction rEference (HIPPIE) database (37) (version 1.4). Interactions with a confidence score of less than 0.7 were removed. The interaction data were then analyzed with the igraph package in R. The interaction data resulted in one large network containing 9,969 nodes and 86 smaller disconnected networks (most with 2 nodes, all contained 7 or fewer) which were removed from the study. The final human PPI network contained 9,969 proteins and 57,615 interactions.

All PPI topology analyses were performed with the R library igraph version 1.0.1 (63).

### Degree adjustment for removal of abundance-degree correlation.

HEK293 cell abundance data from Geiger et al. (64) was used to avoid the effects of differing cell lines. A linear model was fit to the total network’s abundance log_10_ degree data (see Fig. S1A in the supplemental material) using R 3.2.2’s glm function. The correlation slope (0.093) was used to calculate adjusted degree values as follows:

$$\text{adjusted degree}=\left({\text{log}}_{10}\;\text{original degree}-{\text{slope}}_{lm}\times \text{abundance}\right)+{\text{intercept}}_{lm}$$

where lm is the linear model. The final values reported are the 10^adjusted_degree^.

### Statistical analyses and graphics packages.

All statistical tests were performed in R 3.2.2 using the functions prop.test, fisher.test, pairwise.t.test or wilcoxon.test (which performs a Mann-Whitney U test) as appropriate. Prop.test and fisher.test both compare outcome proportions between binomial groups with the latter being more precise for small group sizes. Graphics were produced with either the default graphing features of R or with the ggplot2 library (65).

### Cells and viruses.

Influenza A/WSN/33 virus (WSN) (H1N1) was generated using reverse genetics (66). HEK293 cells were cultured in DMEM (Sigma-Aldrich) supplemented with 10% FCS (10% FCS/DMEM) and antibiotics at 37°C in 5% CO_2_. Virus plaque titers were determined by plaque assay in Madin-Darby canine kidney (MDCK) cells. MDCK cells were cultured in Eagle’s MEM (Gibco) with 5% NCS at 37°C in 5% CO_2_.

### siRNA treatment.

siRNA treatment procedure, cell viability, and virus titer determination are described in detail in Watanabe et al. (32). Briefly, two siRNAs per candidate gene were selected from a predesigned genome-wide human siRNA library (FlexTube siRNA; Qiagen). AllStars Negative Control siRNA (Qiagen) was used as a negative control. The siRNA against the NP gene of WSN virus (GGA UCU UAU UUC UUC GGA GUU) purchased from Sigma-Aldrich was used as a positive control. HEK293 cells were transfected twice with 25 nM (final concentration, 50 nM) siRNA duplexes using RNAiMAX (Invitrogen). At 24 h after the second transfection, cell viability was determined using the CellTiter-Glo assay system (Promega) following the manufacturer’s instructions. To assess influenza virus replication, two parallel sets of siRNA-transfected cells were infected with 50 plaque-forming units (PFU) of WSN virus per well of a 24-well tissue culture plate at 24 h after the second siRNA transfection. At 48 h postinfection, supernatants were harvested, and virus titers were determined by plaque assay in MDCK cells.

### Quantitative reverse transcription-PCR.

To confirm the downregulation of host genes by their respective target siRNAs, quantitative reverse transcription-PCR (qRT-PCR) experiments were performed. Table S6 provides a complete list of primer sequences. HEK 293 cells, transfected twice with 25 nM siRNA (final concentration, 50 nM), were lysed at 48 h posttransfection, and total RNA was extracted by using the Maxwell 16 LEV simplyRNA tissue kit (Promega). Reverse transcription was performed by using ReverTra Ace qPCR RT Master Mix (Toyobo, Osaka, Japan) or SuperScript III reverse transcriptase (Invitrogen). The synthesized cDNA was subjected to quantitative PCR with primers specific for each gene by using the Thunderbird SYBR qPCR Mix (Toyobo). The relative mRNA expression levels of each gene were calculated by the ΔΔ*C_T_* method using beta-actin as an internal control. Primer sequences are available upon request.

10.1128/mBio.02002-18.10 TABLE S6 Primers used for qPCR. Download Table S6, XLSX file, 0.04 MB. Copyright © 2018 Ackerman et al. 2018 Ackerman et al. This content is distributed under the terms of the Creative Commons Attribution 4.0 International license.

### Determining candidate proteins involved in influenza virus replication.

For each set of siRNAs, the virus titers from cells treated with siRNAs were normalized by the titers obtained from cell treated with AllStars Negative Control siRNA (Table S2). siRNAs that reduced cell viability by more than 40% compared to AllStars Negative Control siRNA-treated cells were not considered for further analysis. Unlike our previous study (32), LOESS regression was not needed (Fig. S3). A two-sided, unpaired Student’s *t* test was used to compare the mean virus titers in cells treated with gene-specific siRNAs with those in cells treated with AllStars Negative Control siRNA. Holm’s method for multiple comparisons was then applied to the *P* values.

10.1128/mBio.02002-18.3 FIG S3 Boxplot of the degree and betweenness distributions for connecting (candidate) proteins, virus-interacting proteins, and internal essential proteins. Black lines indicate the median values for the populations. Download FIG S3, TIF file, 0.6 MB. Copyright © 2018 Ackerman et al. 2018 Ackerman et al. This content is distributed under the terms of the Creative Commons Attribution 4.0 International license.

10.1128/mBio.02002-18.4 FIG S4 The mean log fold change (LFC) versus the mean fold change (FC) in cell viability for all 156 gene-specific siRNAs tested. Blue and green points highlight data corresponding to the AllStars Negative Control siRNA and siRNA against influenza virus NP gene (positive control), respectively. The LOESS regression curve (red dotted line) shows that virus growth was not dependent on cell viability. Download FIG S4, TIF file, 0.2 MB. Copyright © 2018 Ackerman et al. 2018 Ackerman et al. This content is distributed under the terms of the Creative Commons Attribution 4.0 International license.
