# Supplementary figures and images for: Network-Guided Discovery of Influenza Virus Replication Host Factors
Source: mBio. 2018 Dec 18;9(6):e02002-18. doi: 10.1128/mBio.02002-18 (PMC6299219; doi:10.1128/mBio.02002-18)

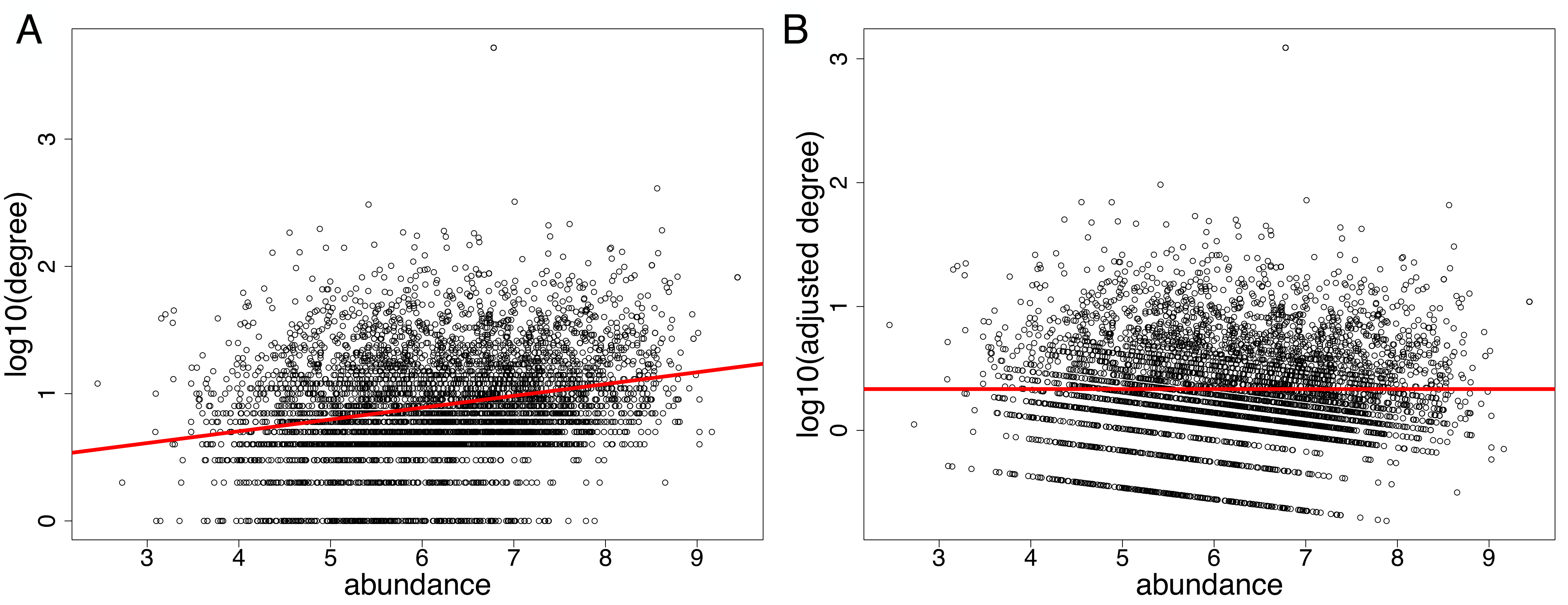

Supplement: FIG S1 [file mbo006184214sf1.tif]

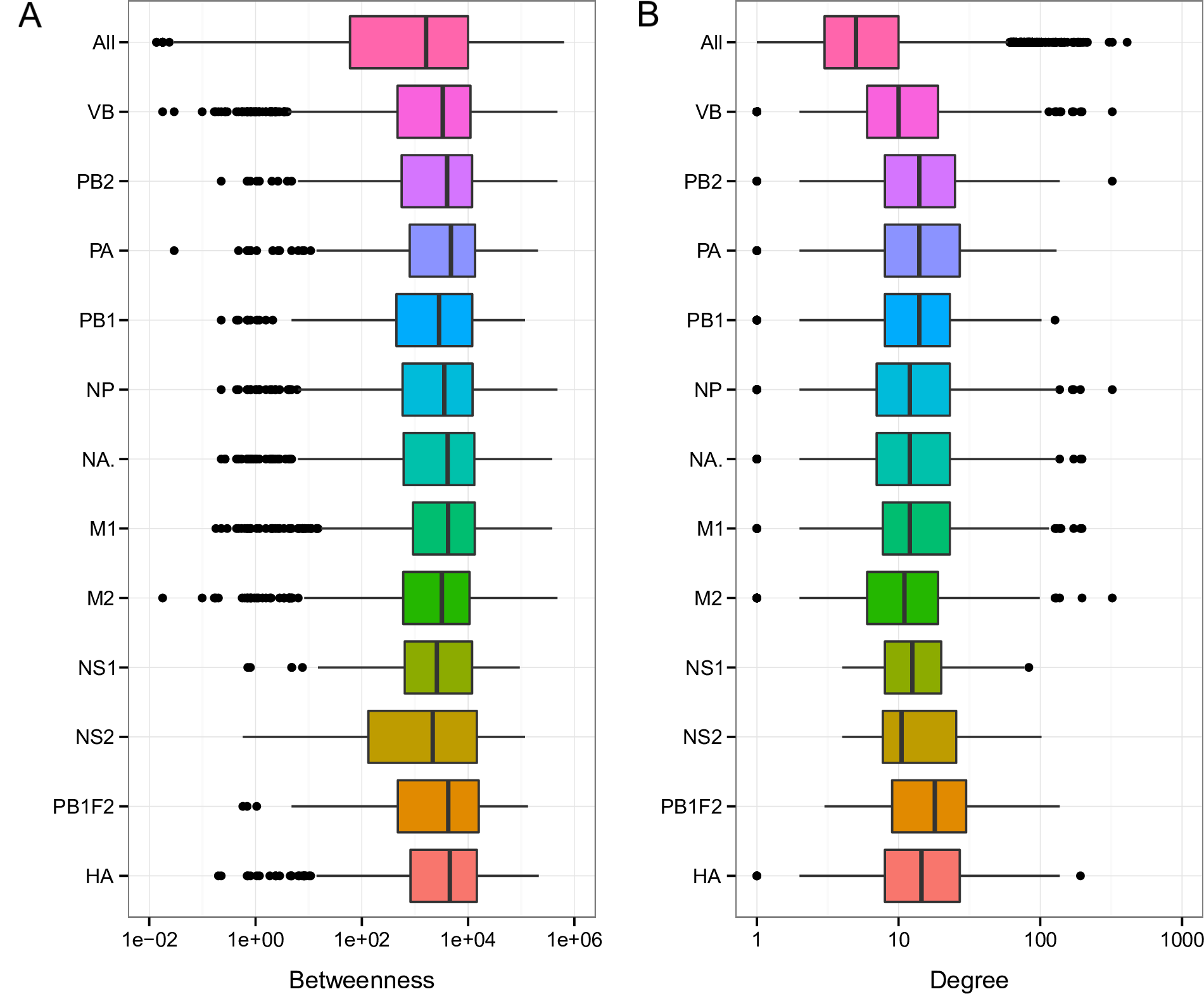

Supplement: FIG S2 [file mbo006184214sf2.tif]

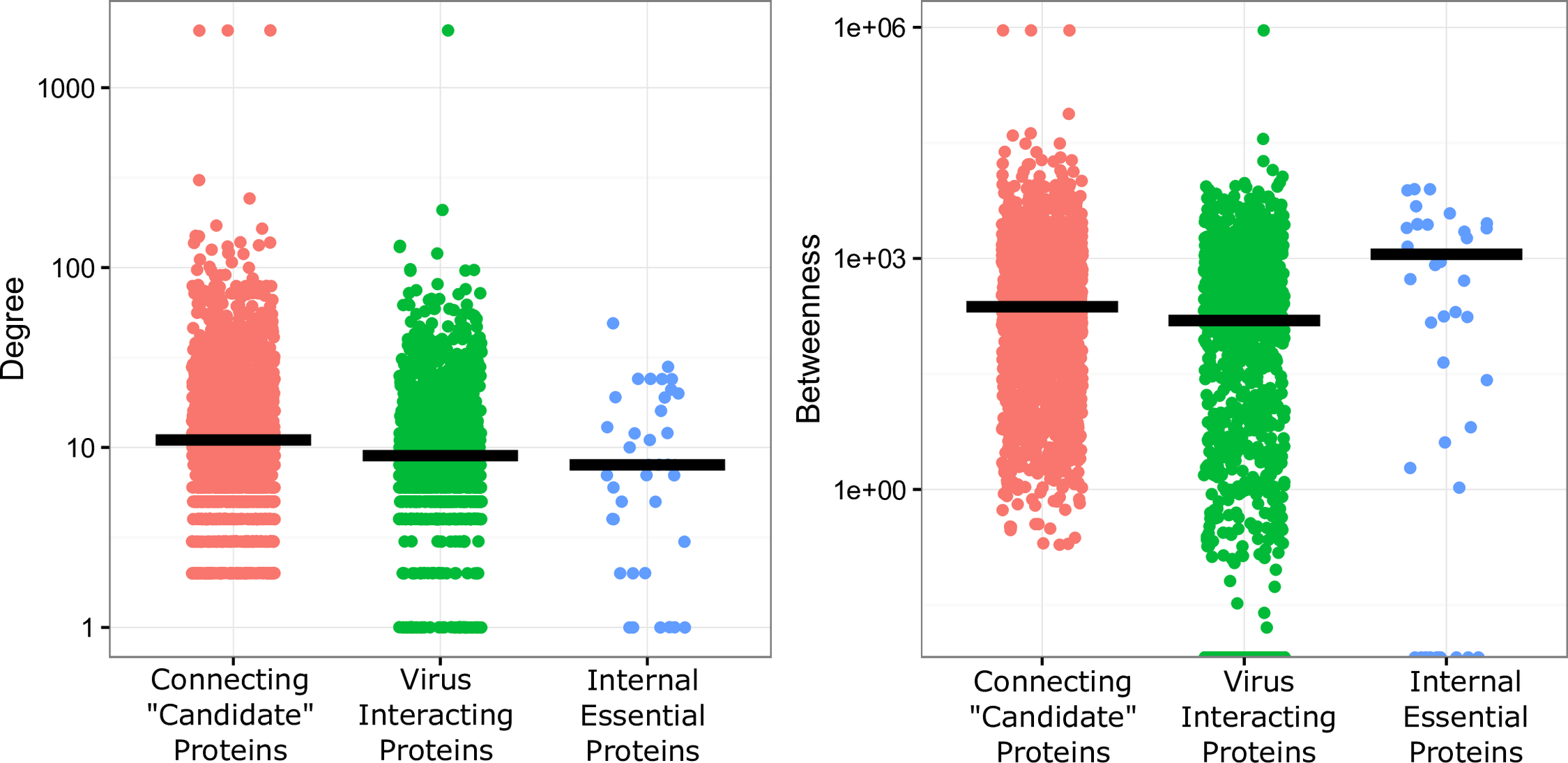

Supplement: FIG S3 [file mbo006184214sf3.tif]

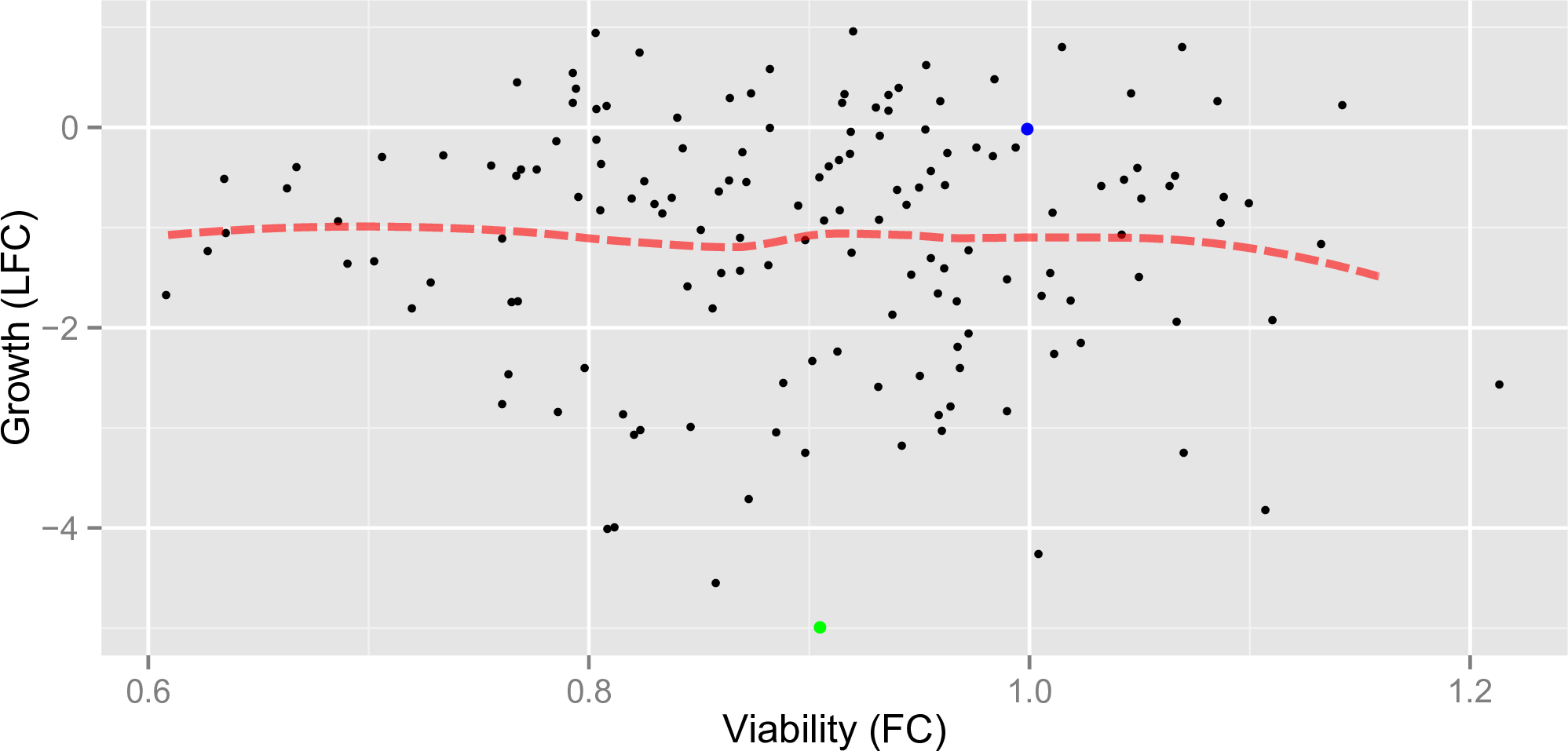

Supplement: FIG S4 [file mbo006184214sf4.tif]
